# Convolutional Neural Networks (CNNs) for Pneumonia Classification on Pediatric Chest Radiographs

**DOI:** 10.7759/cureus.44130

**Published:** 2023-08-25

**Authors:** Yash S Saboo, Saarthak Kapse, Prateek Prasanna

**Affiliations:** 1 Radiology, The University of Texas Health Science Center at San Antonio, San Antonio, USA; 2 Biomedical Informatics, Stony Brook University, Stony Brook, USA

**Keywords:** pneumonia detection, chest x ray, deep learning artificial intelligence, artificial intelligence in radiology, convolutional neural networks (cnn), computer vision

## Abstract

Background: Pneumonia is an infectious disease that is especially harmful to those with weak immune systems, such as children under the age of 5. While radiologists’ diagnosis of pediatric pneumonia on chest radiographs (CXRs) is often accurate, subtle findings can be missed due to the subjective nature of the diagnosis process. Artificial intelligence (AI) techniques, such as convolutional neural networks (CNNs), can help make the process more objective and precise. However, off-the-shelf CNNs may perform poorly if they are not tuned to their appropriate hyperparameters. Our study aimed to identify the CNNs and their hyperparameter combinations (dropout, batch size, and optimizer) that optimize model performance.

Methodology: Sixty models based on five CNNs (VGG 16, VGG 19, DenseNet 121, DenseNet 169, and InceptionResNet V2) and 12 hyperparameter combinations were tested. Adam, Root Mean Squared Propagation (RmsProp), and Mini-Batch Stochastic Gradient Descent (SGD) optimizers were used. Two batch sizes, 32 and 64, were utilized. A dropout rate of either 0.5 or 0.7 was used in all dropout layers. We used a deidentified CXR dataset of 4200 pneumonia (Figure 1a) and 1600 normal images (Figure 1b). Seventy percent of the CXRs in the dataset were used for training the model, 20% were used for validating the model, and 10% were used for testing the model. All CNNs were trained first on the ImageNet dataset. They were then trained, with frozen weights, on the CXR-containing dataset.

Results: Among the 60 models, VGG-19 (dropout of 0.5, batch size of 32, and Adam optimizer) was the most accurate. This model achieved an accuracy of 87.9%. A dropout of 0.5 consistently gave higher accuracy, area under the receiver operating characteristics curve (AUROC), and area under the precision-recall curve (AUPRC) compared to a dropout of 0.7. The CNNs InceptionResNet V2, DenseNet 169, VGG 16, and VGG 19 significantly outperformed the DenseNet121 CNN in accuracy and AUROC. The Adam and RmsProp optimizer had improved AUROC and AUPRC compared to the SGD optimizer. The batch size had no statistically significant effect on model performance.

Conclusion: We recommend using low dropout rates (0.5) and RmsProp or Adam optimizer for pneumonia-detecting CNNs. Additionally, we discourage using the DenseNet121 CNN when other CNNs are available. Finally, the batch size may be set to any value, dependent on computational resources.

## Introduction

Pneumonia is a pulmonary infection where fluid, pus, and/or inflammatory cells fill the air sacs. This significantly reduces the amount of oxygen that dissolves into the bloodstream, making breathing difficult. Common treatments include antibiotics and antiviral medications. Pneumonia kills 2,400 children per day and accounts for 16% of all deaths of children under five years of age, making it the leading cause of death in children [1]. Indeed, two billion people suffer from pneumonia every year [2]. However, treatment can only be adequately started with a proper diagnosis. Chest X-rays (CXRs) can help diagnose pneumonia, especially when there are errors in observing and reporting symptoms in a clinic [3]. However, subtle findings can be missed at times, leading to missed positive cases and unnecessary treatment for negative cases. This problem becomes more pressing as 54% of radiologists report feeling burned out, increasing the chances of a missed finding [4]. Worldwide, a shortage of radiologists disproportionately affects traditionally underserved areas such as rural areas and lower-income countries [5]. AI techniques have proven to be successful in multiple radiological tasks, such as image orientation classification, pathology identification, and abnormality localization [6]. Thus, there is a need to explore how artificial intelligence (AI) can assist in other radiological tasks, such as pneumonia detection. 

Fortunately, AI-driven techniques, particularly deep convolutional neural networks (CNN), are successful in detecting pneumonia on CXRs, with recent models attaining a classification accuracy ranging from 67% to 96% and area under the curve of the receiver operating characteristics graphs (AUROC) ranging from 0.65 to 0.99 [7-11]. Even though CNNs give promising accuracy and AUROC, these models can be further improved through hyperparameter tuning. Similar to how tuning a musical instrument is necessary to create appealing sounds, tuning the hyperparameters of a CNN is vital to improving model performance.

Hyperparameter tuning of pneumonia-detecting CNN models is a relatively under-researched area. This may be because the ideal hyperparameters vary for each CNN architecture and the disease being detected. To the best of our knowledge, there is a limited amount of literature in specifically tuning hyperparameters for pneumonia-classifying CNNs.

Hyperparameters are constant parameters of a CNN that regulate the training process. In an effort to improve CNN’s classification of pneumonia on CXRs, we conducted a hyperparameter tuning test on five state-of-the-art CNNs in order to investigate the effects of architecture complexity and three hyperparameters (optimizer, batch size, and dropout rate) on model performance. Optimizers are algorithms that change the internal weights in a CNN to minimize the CNN’s errors in detecting pneumonia. Batch size is the number of images a CNN must classify before updating its weights. Dropout rate is the percent of neurons that return an output of zero in each layer. We hypothesized that using less complex CNNs, the Adam optimizer, and lower batch sizes and dropouts would result in maximum performance along a variety of metrics.

There has been some, but limited, amount of literature on the effects of architectural complexity on CNN accuracy. Bressem et al. compared 15 CNNs to detect pneumonia and COVID-19 on CXRs and found that CNNs with fewer layers had relatively greater accuracy [12]. However, Bressem et al. did not utilize hyperparameter tuning to optimize each CNN. Instead, each CNN was given the same set of hyperparameters, which decreases comparability between the models because some models benefit from the default set of hyperparameters, while others are put at a disadvantage. If all of the CNNs had been tuned to their own unique set of hyperparameters, a more satisfactory conclusion about the effect of architecture complexity on model performance could be drawn.

There is also a considerable effect of the batch size on model performance. Radiuk tested batch size values from 16 to 1024, in powers of 2, and batch sizes of 50, 100, 150, 200, and 250, on the CIFAR-10 dataset, a dataset of real-world objects, and the MNIST dataset, a dataset of handwritten numbers, and found that the batch size of 1024 was the best performing and 16 was the lowest performing [13]. Bressem et al. verified this finding to medical imaging data. Bressem et al. tested batch sizes of 16 and 32, and concluded that a batch size of 32 gave more accurate results in the detection of 14 pulmonary diseases [12]. It remains to see, however, if such findings will remain true to the specific detection between pneumonia and normal classification. 

The dropout rate is critical to preventing models from overfitting. Overfitting occurs when the model memorizes the CXRs in the training dataset instead of learning generalizable patterns, hindering its performance in classifying pneumonia on unseen data. Dropout sets a certain number of neurons in a layer to have an output of zero. Srivastava et al. found that dropout reduces overfitting in a variety of classification tasks and that the ideal dropout rate, generally, is 0.5 [14]. Dropout reduces the dependence on one set of neurons, and thus, one pattern, because any neuron may randomly be set to zero. The CNN is thus forced to use all neurons in every layer of the model, allowing the CNN to learn a wide variety of cues that indicate the presence of pneumonia. However, an excessively high dropout rate can cause too little information to be passed to the next layer, preventing the model from learning patterns altogether. Thus, too low and too high of a dropout rate could limit the model's generalizability, indicating the need for a dropout rate in the middle of the spectrum. Thus, experimenting with different dropout rates is necessary to find the ideal dropout rate for pneumonia-detecting CNNs.

## Materials and methods

Datasets 

The dataset consisted of 5,863 retrospective, pediatric (one to five years old), anterior-posterior view CXR images taken from Guangzhou Women and Children’s Medical Center, Guangzhou, China. Each CXR image is labeled as either pneumonia or normal by radiologists. CXRs that featured both bacterial and viral pneumonia were excluded from the dataset by the radiologists. CXRs that were low-quality or unreadable were excluded from the study. All CXRs were screened by two expert radiologists for quality and diagnosis. A third expert radiologist was also chosen for checking any diagnosis errors. These CXRs were performed as part of regular patient care. The HIPPA-compliant dataset is free and publicly accessible on the Mendeley Data communal repository [15]. There was no requirement for an Institutional Review Board (IRB) approval as all data are de-identified. In this study, the AI model was not trained to differentiate between bacterial and viral pneumonia.

**Figure 1:**
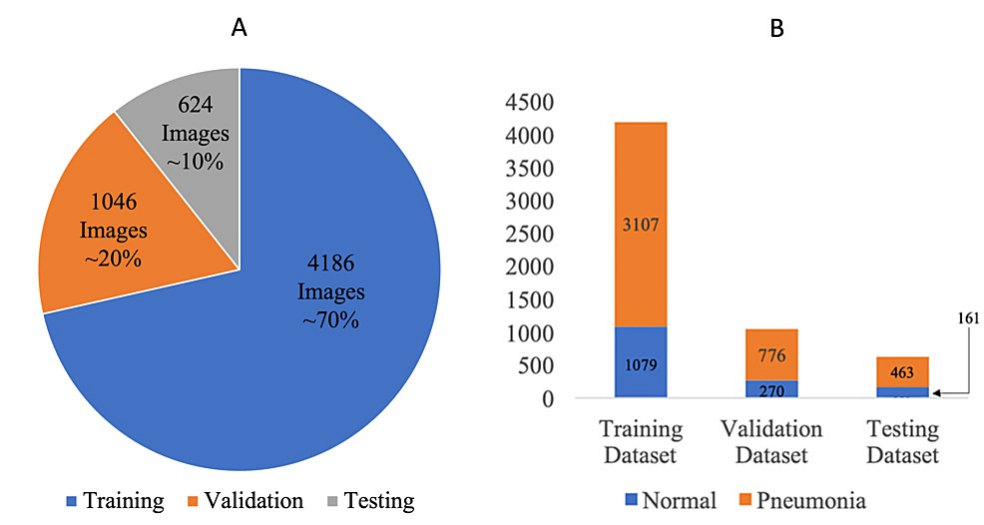
Dataset breakdown into training, validation, and testing sets. A shows the number of images in training, validation, and testing set. B shows the number of pneumonia and normal images in the training, validation, and testing set.

Figure 1 shows that the dataset was split into a 70% training, 20% validation, and 10% testing set, following standard machine learning practices. Random shuffling was used to generate the training, validation, and test set. 

Architectures

Transfer learning is the application of CNNs trained on one dataset, typically consisting of real-world objects, such as the ImageNet’s dataset of handwritten digits, to another dataset, which in this case, are CXRs. Transfer learning was leveraged to minimize computation and training time, as pre-trained CNNs developed from the ImageNet dataset are very generalizable.

Five CNN architectures were chosen on the basis of architecture depth (number of layers). In order of increasing architecture depth, the architectures implemented in this study were VGG 16, VGG 19, DenseNet 121, DenseNet 169, and InceptionResNetV2. After each architecture, MaxPooling Layers, Convolutional Layers, two Dropout Layers, and a Sigmoid Layer were added to fit the CNN to the CXR dataset.

Image pre-processing 

Image pixels were scaled from a range of 0-255 to a range of 0-1 to speed up computation. All images were standardized such that the mean of the pixel values was 0 and the standard deviation was 1. During training, each image was randomly flipped, rotated by 10°, and zoomed in by 10% to create multiple versions of one image. This process augments the training dataset, increasing model performance.

Hyperparameter tuning 

**Figure 2:**
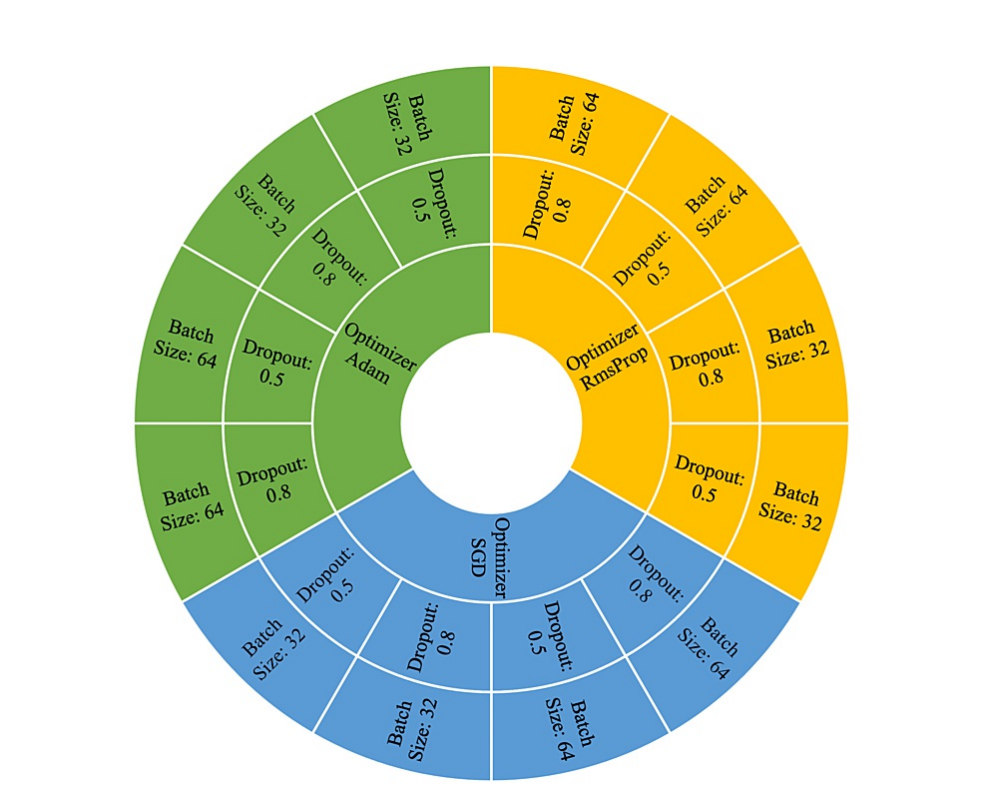
Twelve tested hyperparameter combinations for each CNN. CNN, convolutional neural network

Around 60 models based on five state-of-the-art CNNs (InceptionResNet V2, DenseNet 121, DenseNet 169, VGG 16, and VGG 19) with 12 different hyperparameter combinations were tested, as shown in Figure 2. The hyperparameters of optimizer, dropout rate, and batch size were varied. Three types of optimizer [Stochastic Gradient Descent (SGD), Adam, and Root Mean Squared Propagation (RmsProp)] were tested. Two dropout rates (DP 0.5 and DP 0.8) and two batch size values (32 and 64) were also tested.

Evaluation metrics 

Accuracy (Acc), the area under the curve of the receiver operating characteristics curve (AUROC), the area under the curve of the precision-recall curve (AUPRC), specificity, sensitivity, F1 score, and precision were evaluated for each CNN hyperparameter combination. 

Statistical analysis 

A linear regression test was conducted. The p-value of < 0.05 demonstrated statistical significance. All analyses were performed using the Jamovi statistical software, 3rd Generation. 

## Results

In this study, we investigated three different hyperparameters: optimizer, dropout, and batch size on five CNNs. Three optimizers, two dropout rates, and two batch sizes were used per CNN, leading to 60 different CNN models (5*3*2*2).

**Figure 3:**
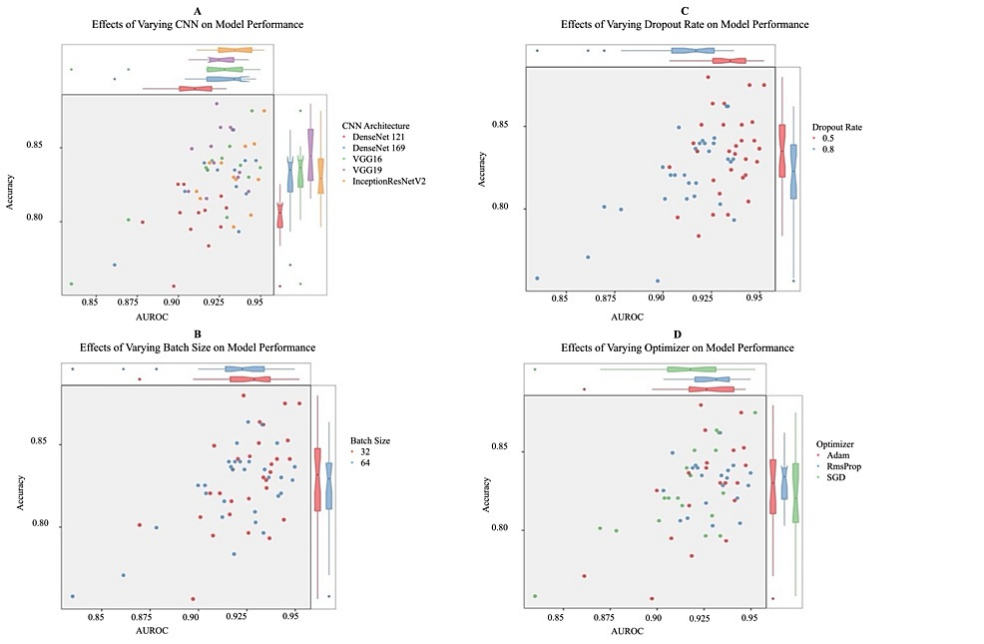
Relating architecture complexity, dropout rate, batch size, and optimizer to accuracy and AUROC. A-D differentiates the 60 models by a certain hyperparameter. A categorizes the data into architectures DenseNet121, DenseNet169, VGG16, VGG19, and InceptionResNetV2. B categorizes the data by batch sizes. C differentiates the data by dropout rate. D differentiates the data by the optimizer. AUROC, area under the receiver operating characteristic curve

Significant results were found with the variation in CNN architectures. As shown in Table 1 and Figure 3A, smaller CNNs (VGG16 and VGG19) had greater test accuracies than larger CNNs (DenseNet121 and DenseNet169). Both shallower and deeper CNNs had comparable test AUROCs. Additionally, linear regression was performed to understand if changes between architectures and hyperparameters are due to random chance. Tables 2-3 indicate that test accuracy, AUROC, F1 score, specificity, and precision indicate statistically significant results (<0.05) when correlated with most CNN architectures. It is crucial to note that a change in CNN architecture had no significant effect on model sensitivity as p values were greater than 0.05.

**Table 1:** AUROC and accuracy of each CNN. AUROC, area under the receiver operating characteristic curve; CNN, convolutional neural network

CNN	Number of layers	Test AUROC	Test accuracy
VGG16	16	0.949	0.875
VGG19	19	0.942	0.879
DenseNet 121	242	0.929	0.825
DenseNet 169	338	0.947	0.862
InceptionResNetV2	449	0.952	0.875

**Table 2:** Linear regression results for varying hyperparameters and CNNs on accuracy, AUROC, AUPRC, and F1 score. CNN, convolutional neural networks; AUROC, area under the receiver operating characteristic curve; AUPRC, area under the precision recall curve; T-value, hypothesis test statistic; p-value, probability value; SGD, stochastic gradient descent; RmsProp, root mean squared propagation

	T-Value accuracy	p-Value accuracy	T-Value AUROC	p-Value AUROC	T-Value AUPRC	p-Value AUPRC	T-Value F1 score	p-Value F1 score
CNN: DenseNet169 – DenseNet121	2.606	0.012	2.179	0.034	2.113	0.040	2.410	0.020
CNN: VGG16 – DenseNet121	2.895	0.006	1.321	0.192	1.545	0.129	2.553	0.014
CNN: VGG19 – DenseNet121	4.531	<0.001	2.320	0.024	3.007	0.004	4.096	< .001
CNN: InceptionResNetV2 – DenseNet121	3.199	0.002	3.572	<0.001	4.597	< .001	2.968	0.005
Dropout Rate 0.8 – 0.5	-2.399	0.020	-5.130	<0.001	-5.280	< .001	-2.782	0.008
Optimizer: RmsProp – Adam	0.426	0.672	0.908	0.368	0.933	0.355	0.472	0.639
Optimizer: SGD – Adam	-0.359	0.721	-2.188	0.033	-3.056	0.004	-0.848	0.400
Batch Size: 64-32	-1.099	0.277	-1.469	0.148	-2.082	0.042	-1.306	0.197

**Table 3:** Linear regression for varying hyperparameters and CNNs on specificity, sensitivity, and precision. CNN, convolutional neural network; T-value, hypothesis test statistic; p-Value, probability value; RmsProp, root mean squared propagation; SGD, stochastic gradient descent

	T-Value specificity	p-Value specificity	T-Value sensitivity	p-Value sensitivity	T-Value precision	p-Value precision
CNN: DenseNet169 – DenseNet121	2.163	0.035	0.1430	0.887	2.225	0.031
CNN: VGG16 – DenseNet121	2.588	0.013	-0.1430	0.887	2.622	0.011
CNN: VGG19 – DenseNet121	4.351	< .001	-0.7152	0.478	4.588	<0.001
CNN: InceptionResNetV2 – DenseNet121	2.725	0.009	0.0613	0.951	2.850	0.006
Dropout Rate 0.8 – 0.5	-0.563	0.576	-2.4683	0.017	-1.048	0.300
Optimizer: RmsProp – Adam	-0.114	0.910	0.7914	0.432	-0.103	0.919
Optimizer: SGD – Adam	0.989	0.327	-2.1209	0.039	0.647	0.521
Batch Size: 64-32	-0.322	0.749	-1.0209	0.312	-0.556	0.580

Hyperparameter tuning was at the crux of this experiment, with variations in dropout and optimizer indicating statistically significant results. Figure 3C shows that the mean AUROC and accuracy for dropout rates of 0.5 are greater than those for dropout rates of 0.8. Tables 2-3 indicate that the dropout rate is statistically correlated with accuracy, AUROC, AUPRC, F1 Score, and sensitivity but not statistically correlated with specificity and precision. There were also some indications of optimizer significance. Linear regression results from Table 3 show that a switch from the SGD to the Adam optimizer had statistically significant improvement on AUROC and AUPRC, but no statistically significant effect on other metrics such as accuracy, F1 score, specificity, and precision. Figure 3D and Tables 2-3 indicate that RmsProp and Adam had similar performance. Additionally, there was almost no statistically significant effect of batch size on model performance, as shown in Figure 3B and Tables 2-3. 

## Discussion

The objective of this experiment is to identify the CNNs and their hyperparameters (dropout, batch size, and optimizer) that best allow for detecting pneumonia on pediatric CXRs. We hypothesized that using the Adam optimizer, less complex CNNs, and lower dropout rates and batch sizes would result in maximum performance. This study found three statistically significant trends in hyperparameter tuning. Specifically, model performance increases with lower architecture complexity, the use of an Adam optimizer compared to an SGD optimizer, and lower dropout rates. Little correlation was found between the batch size and model performance. 

Architecture complexity 

First, less complex architectures (VGG 16 and VGG 19) are better-performing models, based on the majority of metrics (Accuracy, AUROC, AUPRC, F1 Score, Specificity, and Precision). Complex CNNs may be more susceptible to overfitting, or, memorizing the training dataset, instead of learning the general patterns required to detect pneumonia. This, in turn, causes more complex CNNs to have limited generalizability to the testing dataset, decreasing testing accuracy. 

Optimizer 

Second, utilizing the Adam optimizer over the SGD optimizer had a considerable effect in improving AUROC and AUPRC values, although improvement in other metrics was not present. It should be mentioned that no statistically significant difference was found between the Adam and RmsProp optimizers on any evaluation metric. The Adam optimizer may not always be beneficial over the SGD and RmsProp optimizer, but it may occasionally benefit model performance. 

Dropout 

Third, a lower dropout rate is significantly correlated with increased model performance through an increase in most metrics (Accuracy, AUROC, AUPRC, F1 Score, Sensitivity). This may be because the higher dropout rate of 0.8 caused information loss in the CNN, thereby hindering model learning. 

Comparison to other studies 

Many studies comparing CNN architectures to detect pneumonia do report hyperparameters but did not engage in hyperparameter tuning. For example, Rahman et al. and Toğaçar et al. used a batch size of 16 and SGD optimizer but did not vary the batch size and optimizer to improve model performance due to limited memory usage and time constraints [7, 16]. Hashmi et al. also used an SGD optimizer but did not report batch size values nor engage in hyperparameter tuning [17]. Ayan and Ünver followed a similar practice, developing a CNN with 87% accuracy, but arbitrarily selecting the RmsProp optimizer [18]. Saraiva et al. reported a 94% accuracy but failed to report any hyperparameter values [19]. The lack of hyperparameter tuning within recent work suggests that there is a window to improve model performance.

It is important to note that Rahman et al., Toğaçar et al., Hashmi et al., and Saravia et al. developed CNNs that outperform the models in this study in terms of accuracy and AUROC [7, 16-17, 19]. Our model may have underperformed due to a lack of certain image pre-processing routines and the lack of k-fold cross-validation. There may have also been uncontrollable factors at play, such as the random initialization of the CNNs weights and random variation in gradient descent.

This study still has significant value because of the correlation found between dropout rate, optimizer, and architecture complexity on CNN model performance. Since the Adam optimizer results in higher AUROC and AUPRC values than the SGD optimizer, it is of interest to replace the SGD optimizer with an Adam optimizer on existing CNN models. Additionally, further results may look to using lower dropout rates to enhance state-of-the-art models. 

Model performance is also dependent on the CNN architecture that is being utilized. While there has been significant discussion about the optimal CNN architecture in detecting pneumonia, most work has not accounted for hyperparameter tuning. By comparing 15 different CNNs, Bressem et al. find that CNNs with fewer layers perform better than larger-scale CNNs [12]. Ayan and Ünver confirm this result by finding that VGG16, a 16-layered network, performs better in classifying pneumonia than Xception, a large-scale 71-layer network [18]. However, these studies' methodologies may be improved if the CNN's hyperparameters were tuned. Our novel study compares hyperparameter-tuned CNNs to allow for fairer, more comparable results because the correct set of hyperparameters can specialize the CNN to the task. Our results align with those of Bressem et al. and Ayan and Ünver, as our study finds that less complex CNNs (VGG 16 and VGG 19) performed comparatively better than more complex CNNs (DenseNet 169 and InceptionResNet V2). The highest-performing CNN architectures shown in Table 1 may be recommended to hospitals pursuing AI capabilities in radiology. 

Our results indicate that there is little necessity for very deep CNNs, with hundreds of layers, in order to classify pneumonia. This finding allows for lower hardware requirements to employ the model in clinical practice, incentivizing greater usage of CNNs in screening facilities.

Limitations

This study’s findings could be strengthened by increasing the sample size of CNN architectures, dropout rates, batch sizes, and optimizers. The suggested hyperparameters should be applied to a CNN model to classify a variety of diseases, including atelectasis, cardiomegaly, edema, and pleural effusion in order to verify the generalizability of the hyperparameters.

## Conclusions

The VGG-19 model, characterized by low dropout rates (0.5), less architecture complexity, and an Adam optimizer serves as a compelling CNN model for use in radiological settings for the optimal detection of pneumonia on pediatric CXRs, achieving an accuracy of 87.9%. The batch size had no statistically significant effect on model performance. The low dropout values, low architecture complexity, and use of an Adam optimizer serve as recommendations in the hyperparameter tuning process of CNNs to classify pediatric pneumonia. 
